# The New Panel Agreements

**Published:** 1919-11-22

**Authors:** 


### THE NEW PANEL AGREEMENTS.

It has been made public that proposals for various alterations of the existing agreements under which panel doctors work have been put forward by the Ministry of Health, and are now under consideration by the Panel Committees. Although the precise wording of these suggested amendments is not available at the moment of writing, a synopsis of what they involve has appeared, from which their scope can be fairly well adjudged. These negotiations, it is safe to infer, mean that various complaints made against panel doctors, individually or in the bulk, and against the panel system have been accepted by the Ministry of Health as well founded. It is far from our intention to hold up panel practitioners as paragons: to put it bluntly, all the "scallywags" in the medical profession are on the panels, and they sometimes swamp the more conscientious and better educated types. But at the same time, it is not fair to ascribe all, or nearly all, the justifiable complaints against the panel service to the faults of individuals rather than of the system under which they work. The conscientious worker gets little encouragement: the shirker too often flourishes under it.

Granting, then, that the system badly needs overhauling, it is yet permissible to doubt whether the Ministry of Health is right in its remedy of further harassing the panel practitioner. The new proposals include provisions that he may not go away upon a holiday without informing the Insurance Committee of those responsible for doing the work in his absence: not an unreasonable stipulation in itself, perhaps, but dangerous if it presages attempts by Insurance Committees (not usually ideal employers to work under) to dictate when a doctor shall have a holiday, how much he shall have, and to whom he shall delegate his responsibilities. Again, the panel doctor is not to employ an assistant without first getting the Committee's consent: this, too, reasonable enough with a sensible committee, but likely to be an instrument of tyranny in the hands of certain types of those bodies which are not uncommon. It is also laid down that he shall keep such records of the diseases of his patients and of his treatment of them in such form as the Ministry may from time to time determine. Well persuaded of the value of systematic case-taking, we are yet convinced that this will result in incessant worrying of the practitioner without any resultant advantage to his patients, simply to provide the Department with an unfailing supply of "eyewash." Of the same purport, it may well be suspected, is the proposed regulation requiring reports on diseases, which may be asked for by any order made by the Minister.

These new regulations appear thoroughly bureaucratic in inspiration. By which adjective is not meant that they are necessarily bad in themselves, but only that they betray unfamiliarity with life as it confronts men practically. The man in the office armchair is apt to think that every difficulty outside can be solved by issuing the appropriate form for somebody else to fill up in duplicate, and for clerks to file when it is returned to the office. The panel service is admittedly bad: probably worse than even the Ministry of Health suspects. But much of its badness arises from causes inherent in the scheme itself, and duly pointed out when the Insurance Act was being debated: more still arises from those evils which State control seems inevitably to bring in its train. The new proposals seem more likely to intensify the evils than to cure them.

